# Knowledge, attitudes, and acceptability of direct-acting antiviral hepatitis C treatment among people incarcerated in jail: A qualitative study

**DOI:** 10.1371/journal.pone.0242623

**Published:** 2020-12-02

**Authors:** Matthew J. Akiyama, Jonathan Ross, Fatimah Rimawi, Aaron Fox, Alison O. Jordan, Janet Wiersema, Alain H. Litwin, Fatos Kaba, Ross MacDonald

**Affiliations:** 1 Department of Medicine, Montefiore Medical Center/Albert Einstein College of Medicine, Bronx, NY, United States of America; 2 Correctional Health Services, New York City Health + Hospitals, New York, NY, United States of America; 3 Department of Medicine, University of South Carolina School of Medicine–Greenville, Greenville, South Carolina, United States of America; 4 Department of Medicine, Prisma Health, Greenville, South Carolina, United States of America; 5 Clemson University School of Health Research, Clemson, South Carolina, United States of America; University of North Carolina at Chapel Hill, UNITED STATES

## Abstract

**Introduction:**

While U.S. jails are critical sites for engagement in HCV care, short lengths-of-stay often do not permit treatment in jail. Therefore, linkage to HCV care after incarceration is crucial. However, little is known about HCV treatment acceptability among justice-involved individuals in U.S. jails. The goal of this study was to understand knowledge, attitudes, and acceptability of HCV treatment among people living with HCV in the New York City (NYC) jail system.

**Methods:**

We recruited 36 HCV-antibody-positive individuals in the NYC jails using clinical data reports and performed semi-structured interviews to explore participants’ attitudes toward HCV treatment in jail and following return to the community. We continued interviews until reaching thematic saturation and analyzed interviews using an inductive, thematic approach.

**Results:**

Participants were mostly male, Latina/o, with a mean age of 40 years. Nearly all were aware they were HCV antibody-positive. Two thirds of participants had some awareness of the availability of new HCV therapies. Key themes included: 1) variable knowledge of new HCV therapies affecting attitudes toward HCV treatment, 2) the importance of other incarcerated individuals in communicating HCV-related knowledge, 3) vulnerability during incarceration and fear of treatment interruption, 4) concern for relapse to active drug use and HCV reinfection, 5) competing priorities (such as other medical comorbidities, ongoing substance use, and housing), 6) social support and the importance of family.

**Conclusions:**

Patient-centered approaches to increase treatment uptake in jail settings should focus on promoting HCV-related knowledge including leveraging peers for knowledge dissemination. In addition, transitional care programs should ensure people living with HCV in jail have tailored discharge plans focused on competing priorities such as housing instability, social support, and treatment of substance use disorders.

## Introduction

Hepatitis C virus (HCV) is the leading cause of cirrhosis, liver failure, and hepatocellular carcinoma in the United States (U.S.) [1, 2]. The majority of HCV infections in the U.S. are due to injection drug use (IDU) [3]. Given the interrelationship between IDU and incarceration, HCV prevalence in justice-involved populations is approximately 10–20 times higher than in the general population [4]. Due to the high HCV prevalence in a setting that provides relative stability from active drug use, the criminal justice system has been recognized as an essential component for HCV elimination strategies [5].

The U.S. has the highest rate of incarceration worldwide. At the end of 2016, there were nearly 2.2 million people in prison or jail with an incarceration rate of 860 per 100,000 adults ages 18 and older [6]. Unlike prisons, which house convicted individuals whose sentences are typically greater than one year, jails are shorter-term facilities with a median length of stay of approximately 7 days [7]; however, lengths of stay are unpredictable and may last several weeks to months while a detainee is undergoing or awaiting trial. There are nearly 11 million admissions to U.S. jails per year, which is approximately eighteen times the number in state and federal prisons [8, 9]. Due to the high volume of people living with HCV cycling through them, jails are essential to HCV elimination in the U.S.

With the emergence of short-course, highly effective, and well-tolerated direct-acting antiviral (DAA) therapy, jail-based treatment is feasible for a subset of people living with HCV [10]. If DAA therapy can be delivered prior to release or transfer, corrections-based HCV treatment is equivalent to community-based treatment [11]. However, given the rapid turnover in jail settings, many people living with HCV cannot be treated prior to release; therefore, linkage to HCV care after incarceration is crucial.

Available data from public health programs and pilot studies suggest low rates of linkage to HCV care after incarceration in jail [12–14]. During incarceration people experience social, structural, and medical disruptions. These can lead to poor social support, unstable housing, lack of access to health insurance, and exacerbation of mental health conditions upon return to the community [15–19]. The re-entry period is also associated with an increase in active substance use, carrying a theoretical risk for HCV transmission [20–23]. Therefore, linkage to HCV care is a high priority both for individual health outcomes and to prevent forward HCV transmission.

While barriers to and facilitators of HCV treatment with DAA therapy in prison settings have recently been described [24], to-date little is known about the attitudes of incarcerated individuals in short-term jail settings toward HCV treatment. Successful HCV elimination strategies require data from patients’ perspectives to inform interventions that encourage treatment uptake while in jail, and linkage to HCV care post-incarceration. The goal of this study was to improve our understanding of knowledge, attitudes, and acceptability of HCV treatment among incarcerated people living with HCV in the New York City (NYC) jail system.

## Methods

### Setting

We conducted semi-structured, qualitative interviews with 36 HCV antibody-positive individuals in NYC jails between March and April 2015. The NYC jail system is the second largest in the U.S. with nearly 50,000 admissions per year and an average daily census of almost 9,000 as of early 2018 [25]. Approximately 25% of jail incarcerations have a duration of less than 72 hours. Roughly two-thirds of jail incarcerations result in return to the community; with one quarter to one third of those released being subsequently re-incarcerated [25, 26]. In 2013–2014, the HCV antibody positivity prevalence based on risk factor and birth cohort screening in the NYC jails was 21% [27]. At the time the study was conducted, HCV treatment was being offered in the NYC jails for those whose lengths of stay was sufficiently long to complete treatment in jail.

### Recruitment

We used clinical data reports to identify potential participants who were HCV antibody-positive at one male and one female facility on Rikers Island. We then invited these individuals to the jail clinic for HCV counselling on a voluntary basis. Regardless or participation, participants were offered standard HCV services while in the clinic including counseling, testing, and treatment, if eligible. While in clinic, an investigator (MJA) offered participation in the study, which was explained in detail and it was made clear that opting out would not have any impact on medical care. Participants were not compensated for participating in the study. If potential participants agreed, written informed consent was obtained. Inclusion criteria were: 1) HCV antibody positivity; 2) 18 years of age or older; and 3) fluent in English or Spanish. Individuals with prior HCV treatment were not excluded from the study. Sampling was by convenience, and we purposefully targeted participants of variable age, sex, and race/ethnicity. The study team obtained written informed consent from all participants. Approval to conduct the study was obtained from the NYC Department of Health and Mental Hygiene Institutional Review Board.

### Interviews

An interview guide was developed by three investigators (MJA, FK, RM) to elicit participants’ knowledge of HCV and HCV treatment as well as the acceptability of HCV treatment among jail detainees. For example, to elicit knowledge of HCV, participants were asked: “Please tell me what you know about hepatitis C.” To explore the acceptability of HCV treatment in jail, participants were asked: “How would the feel about initiating treatment in jail?” After obtaining written informed consent, one-hour face-to-face interviews were conducted in private clinic areas in the NYC jails by a male infectious diseases physician with training in medical anthropology and clinical research and had no prior relationship with participants prior to initiating the study (MJA). The interviewer made field notes following interviews regarding interview completion, reliability, and inconsistencies in responses. We conducted interviews until we achieved thematic saturation. All interviews were conducted in English, audiotaped and professionally transcribed. After transcription, transcripts were deidentified for analysis.

### Analysis

We analyzed the data in an iterative process using a process of inductive thematic analysis [28]. Three investigators (MJA, JR, ADF) developed a coding scheme to categorize common themes that we identified inductively upon iterative readings of the first five transcripts. This coding list was then applied to all 36 transcripts with two investigators (JR, FR) independently coding each one. Agreed-upon codes were entered into Dedoose (Version 8.0.35, Los Angeles, California) so that content from all transcripts could be sorted and extracted by code. After coding all transcripts, investigators reviewed excerpts, examining themes within codes as well as between codes, and used the constant comparative method to identify, refine and consolidate emergent themes [29]. The research team then discussed transcripts and discrepancies in coding or revisions to the coding list were resolved by consensus. All cited excerpts are identified by the participant’s age and sex. These findings have been reported in accordance with COREQ guidelines [30].

## Results

Participants were mostly male (n = 21) and Latina/o (n = 17), and had a mean age of 40 years. Few were high school graduates (n = 13). The probable HCV risk factor for the majority of study participants, based on its strong epidemiologic link with HCV, was IDU (n = 28). Mean ages of initial reported drug use and IDU were 15 and 25, respectively. Mean ages of first arrest and incarceration were 26 and 28, respectively (Table 1).

**Table 1:** Self-reported sociodemographic characteristics of study participants.

Characteristic	N = 36 (%)
Age, mean years (SD)	40 (10)
Female	15 (42)
Race/ethnicity	
Latina/o	19 (53)
Caucasian	9 (25)
Black	7 (19)
Asian	1 (3)
High school graduate	13 (36)
HCV risk factor	
IDU	28 (78)
INDU	6 (17)
Other	2 (5)
Mean (SD) age of:	
First drug use	15 (5)
First injection	25 (9)
First arrest	26 (10)
First incarceration	28 (11)
Aware of HCV antibody status/exposure prior to enrollment	32 (89)
Aware of new HCV therapy	22 (61)

HCV = Hepatitis C virus, IDU = Injection drug use, INDU = intranasal drug use.

Broadly, jail emerged as an important location to engage incarcerated persons regarding HCV treatment however, the jail environment itself presented several barriers. Key themes included: 1) variable knowledge of new HCV therapies affecting attitudes toward HCV treatment, 2) the importance of other incarcerated persons in communicating HCV-related knowledge, 3) vulnerability during incarceration and fear of treatment interruption, 4) concern for relapse to active drug use and HCV reinfection, 5) competing priorities (such as other medical comorbidities, ongoing substance use, and housing), 6) social support and the importance of family.

### Variable knowledge of new HCV therapies affecting attitudes toward HCV treatment

Most participants were aware they had been exposed to HCV or were HCV antibody-positive (n = 32) and nearly two-thirds of participants had some awareness of the availability of newer HCV treatment (n = 22), even if they had not heard specifically about DAAs. Those aware of DAAs or some of their characteristics reported being more inclined to start HCV treatment. Aspects of DAA therapy (e.g., short duration, few adverse effects, no injections) made them acceptable, and concerns about worsening health led to favorable attitudes toward HCV treatment to prevent further health deterioration. However, there were also concerns about adverse effects stemming from poor knowledge about DAAs and concerns about potential side effects.

An HIV/HCV co-infected participant with antiretroviral treatment fatigue felt that DAA therapy would be less problematic compared to interferon-based therapy:

“I just, I got tired of taking them [ARVs]. It’s [DAA therapy] just not as long. It’s not as long a period… they’re not as fierce. There’s less depression. They’re not as harsh.” (53-year-old male)

Another participant expressed interest in HCV treatment not involving injections because they were a trigger with respect to relapse to IDU for her:

“Yeah. Pills. No injections. I don't want any needles. I'm done with that. I'm healed and everything, I'm good. It's just a trigger. I don't want to use those anymore.” (22-year-old female)

Several participants expressed interest in HCV treatment due to a feeling their health was currently poor or for the positive health benefits such as this participant:

“It’s getting to be that time. I need to. Because I’m getting sick. Fatigue, you know, all that. No energy.” (40-year-old male)

Others wanted HCV treatment because they were tired of drug use and HCV treatment represented a new start.

“I want to stay away from negativity. I’m serious about it [HCV treatment]. That’s why I stopped using drugs. And I think the more that I don’t use drugs I think I want to get myself clean.” (44-year-old male)

While overall support for HCV treatment was high, some had concerns regarding adverse effects such as this participant:

“Of course. Anytime you start [hepatitis C] treatment. That's like taking chemo for cancer.” (47-year-old female)

Others were aware of newer HCV treatment, but without sufficient knowledge, they were reluctant to actively seek treatment.

“The hesitation just stems from I don’t really have a lot of knowledge about it. It’s something that I would obviously be interested in because it might save my life. I heard it’s a bad liver disease and I know that depending on what the levels are, depends how sick you could be from it. But I’ve also heard that there’s a new treatment that came out that might be something that I could do.” (35-year-old male)

### The importance of other incarcerated individuals in communicating HCV-related knowledge

A recurrent theme about the way in which HCV-related knowledge was acquired was through communication with other incarcerated individuals. Many conveyed they had received a considerable amount of information about HCV from other incarcerated individuals in jail.

“I heard that yesterday from my friend that sits next to me. That’s the first time I heard of there being types of hepatitis C. To me it was the same shit. I know that there was levels and it would determine how sick you are, but I didn’t know about types.” (56-year-old male)

Similar to HCV-related knowledge; communication with other incarcerated individuals regarding knowledge of HCV treatment appeared to be a key source of information for study participants.

“I had a roommate not too long ago. He’s Hep C, and was telling me the treatment and he said he take medication for it, and he said yo, get the treatment. You know what I mean? I said okay. I go get the treatment, and he said, yo just get the treatment. Whatever you do, get the treatment. Don’t, don’t just leave it like it is, man.” (41-year-old male)

### Vulnerability during incarceration and fear of treatment interruption

Most participants reported they would be interested in starting HCV treatment while in jail. However, some participants expressed specific concerns. Many were rooted in feeling vulnerable and physically weak from treatment resulting in an inability to protect themselves while in jail.

“The only concern I have is what the treatment entails—if I’m going to be sick from it; if I’m going to be weak from it. Those are things I need to worry about because I’m in an environment that I can’t really depend on right now. I gotta have an awareness that’s different from the street. I’m in a place that’s full of criminals from thieves to murderers to whatever.” (56-year-old male)

When asked if they would rather wait as opposed to initiating treatment while incarcerated participants raised concerns about treatment interruption and fear of resistance.

“I don't want to have to start over. You know what I mean? I don't know if it's true, but with antibiotics or anything, antiretrovirals, if you start and then stop, your body can become resistant to the drug. And you might have to do something that's more a rigorous regimen and I just don't want to do that.” (22-year-old female)

One participant who had an HCV treatment interruption noted the importance of her healthcare provider and her family in minimizing treatment interruption when she was incarcerated and upon return to the community.

“My doctor gave a me a letter saying all the medication I was on. I gave them the information so I got medicated quick. I missed two here and two at home. So I missed four days of the Hep C medication. Because I was going through the [jail] system. But I have my medication at home. I was good about my own situation. My whole family knows and I got good family support. My doctor calls my family. I've got good family.” (48-year-old female)

### Concern for relapse to active drug use and HCV reinfection

Some participants described how substance use and relapse, including the risk of reinfection, were important factors they felt would deter them from engaging in HCV care. In certain cases, this led to the conclusion to avoid HCV treatment for the time being.

“It would be stupid on my part to go through a process like that and then screw it all up. If I made a decision to do that I would keep being risk free after that. You know what I mean? That just wouldn’t make sense. This is probably why I haven’t pursued it so far. I know for myself that I have it [hepatitis C], but since I’m using heroin and self-medicating I’m just keeping it to the side for now. I know if I had to confront it at some point and deal with it. I’m going to want to do it properly.” (38-year-old male)

This was mirrored among some participants whose knowledge of their HCV status had already led to reported behavior changes when in the community to avoid forward HCV transmission, such as not sharing needles and having safer sex. One participant who was HCV-positive when she relapsed after being in a drug treatment program recalled that she had avoided sharing injection equipment because of what she had learned in the program: “'Cause I knew more about it, yeah.” (33-year-old female).

### Competing priorities perceived to complicate linkage to HCV care after incarceration

Factors other than substance use that are known to complicate linkage to care after incarceration are unstable housing and unemployment [31]. Describing the interplay of housing instability, unemployment, and substance use competing with his motivation to seek HCV treatment one participant noted:

“Actually, I was kind of like, you know, homeless. Trying to work and trying to keep active, see if I can get a job and my own apartment and that’s all I been fighting with. But me getting sick, I haven’t. Drug use, that’s all that kept me in motion.” (45-year-old male)

Additional competing priorities perceived to complicate linkage to HCV care included other chronic medical and legal difficulties.

“I’m going through so much shit. Right now, I got a little cold. And I know this treatment that it’s going to affect me, because my T-cells is really low. Not now. I’m not ready for it. I believe that when I get that treatment, they’re going to put me in bed… So I prefer to get back in three or four hundred T-cell and then go for the treatment. But for now, I don’t want to take a chance. Especially here, they treat you like shit. No, no, no. Also I got a legal problem, man, if I feel sick and I get sick. No, I ain’t going for that now. (42-year-old male)

Factors that participants perceived to mitigate some of these barriers and might aid in linkage to HCV care included having an existing physician in a health system to return to. Participants frequently alluded to the importance of integrated healthcare and substance treatment services.

“I plan on seeing my primary doctor. I'm in a methadone program. And they have a primary doctor that sees everyone and since they know about my case and everything, I'm more apt to take, you know, the medication there.” (47-year-old female)

### Social support and importance of family

For many participants, family was a key motivator driving their interest in HCV treatment. One participant’s desire to be healthy for his family when he is released drove his interest in initiating treatment while incarcerated:

“Once I heard I had hepatitis C, I was like, you know, this is a disease and ain’t no cure for it. You know what I’m saying. So, I wouldn’t mind. I wouldn’t mind seeking treatment [in jail], but—if, you know, there’s a cure… You know, I have five daughters and now I got three grandkids. So, now I hope—I want to stay alive a little longer. Got some hope… I got grandkids I said … I’d love to be with them.” (45-year-old male)

In contrast, for some, the absence of family or loved ones impaired self-care and presented a barrier to HCV treatment uptake.

“When you don’t feel [loved] then you don’t care. And that’s how I felt when my mother died. And that’s what kept me on that stroll. I didn’t care about nothing or nobody. Just getting high so I didn’t have to think about it.” (42-year-old female)

## Discussion

To our knowledge, this is the first study examining incarcerated individuals’ knowledge, attitudes, and acceptability of HCV treatment in a short-term U.S. correctional setting. In contrast with other studies in correctional settings in which stigma leads to a reduction in disclosing information about one’s diagnosis [24, 32], communication between incarcerated individuals appeared to be a key feature in acquiring HCV-related knowledge. Like other studies in correctional settings, we identified gaps in participant knowledge regarding their HCV status and features of DAA therapy, which could serve as barriers to HCV care signaling a need for improved testing and education programs [33]. While HCV treatment was viewed favorably by most, we also elucidated reasons why participants may choose to forgo HCV treatment during incarceration. Concerns that adverse effects from medication would increase vulnerability in jail; disruptions in treatment could lead to resistance; or lack of readiness for treatment due to competing priorities like legal problems, unstable housing, or ongoing substance use were also common.

Most participants in our study viewed treatment initiation in jail favorably, mirroring findings from other studies in which correctional settings were perceived as a time of stability and access to healthcare [32, 33]. In our study, however, we demonstrate DAA therapy-related concerns specific to the jail setting such as treatment interruption and the risk for viral resistance. Further investigation is warranted into the structural-level factors that contribute to treatment interruption for those entering jail on DAAs and for those continuing them post-incarceration, and how to mitigate them. Examples may include structured questions regarding community DAA therapy at intake, assessing projected lengths of stay, considering medical holds prior to transfer to other facilities, such as prison, to complete treatment in jail, clinics that span jail and community settings, or issuing take-home medication at the time of release [11]. In addition, measures should be taken to ensure continuity of care between the correctional setting and community providers including culturally-competent transitions clinics and patient navigation interventions [34, 35].

Structured assessments of HCV treatment readiness focus on motivation, self-efficacy, social support and stability, alcohol and substance use, among others [36]. For people in correctional settings, recent substance use, housing instability, and social isolation highlight challenges to treatment readiness during and after incarceration. However, some participants also saw their jail stay as a time to consider life changes, including HCV treatment initiation as a way to prioritize their health for themselves and their family. Other research has also shown that people in prison are motivated to undergo HCV treatment both for their own personal health and as a gesture of reciprocity to loved ones in the community [37]. For many participants in our study, HCV treatment represented ‘a new start.’ Such personal transformation associated with HCV therapy have been noted elsewhere in correctional settings in both the interferon and DAA eras [24, 33]. Regardless, structured models of post-release linkage to HCV care with case management and other support will be necessary to assure good HCV treatment outcomes.

Like other studies [24, 32, 33], HCV knowledge gaps affected attitudes toward treatment among our participants. We determined that knowledge of DAA therapy tolerability and efficacy should also be emphasized. Participants in our study who were aware of new HCV treatment were eager to initiate on it. However, fear of HCV treatment was evident for those unaware; likely a ‘carry over’ from the interferon-era. An interferon-era study in the Australian prison system found that people incarcerated in prison feared interferon injections, adverse effects, vulnerability related to physical weakness, and relapse to IDU as a consequence of treatment injections [32]. Many of these perceptions overlapped with those observed in our study.

Another cited barrier to treatment in correctional settings is a concern regarding high HCV prevalence and the risk of reinfection [24, 32]. Reinfection due to ongoing IDU and prioritizing substance use in general diminished enthusiasm for HCV treatment among our participants. Persons who are incarcerated with substance use disorders should have access to standard of care treatments such as opioid agonist therapy and harm reduction services [38].

Competing priorities beyond substance use disorders, such as housing, unemployment, medical comorbidities, and legal difficulties were all anticipated barriers to linkage to care. Interventions including co-location of services in the community to alleviate the need for travel to many locations for care should be implemented so that substance use disorders, other comorbidities, and social determinants of health are addressed in the transition to the community [24, 33, 39].

In other settings, a loss of confidentiality and stigma were identified barriers to treatment uptake in prison [24, 33]. Participants of this study communicated variable degrees of perceived stigma with regard to their HCV status. We do not address stigma specifically in this manuscript because it was not mentioned as a barrier to treatment either in the correctional setting or upon return to the community. In contrast, many participants reported receiving knowledge from other incarcerated individuals that enriched their understanding of their diagnosis. In recent studies, the integration of peers into corrections-based care has improved knowledge, reduced risk-taking behaviors, and strengthened healthcare service engagement by reducing fear, stigma and encouraging mutual trust [40, 41]. This relationship should be explored further and may support the role of corrections-based peers in HCV education and linkage to care.

Social support after incarceration has been demonstrated to facilitate linkage to HCV care when present and hinder when absent [24, 34, 42]. Our study participants expressed views that reinforce these findings particularly with regard to family; participants wanted to get well for their family and people who felt isolated had ‘given up’ on caring for themselves. Because many justice-involved individuals experience disruptions in their interpersonal relationships, support from case managers and patient navigators (peers or otherwise) may be crucial to facilitate linkage to HCV care.

This study has several limitations. There is a risk of social desirability bias since it was conducted in a correctional setting [43]. However, participants were informed that their responses were confidential and would not affect the nature of their care in any way. Interviews were conducted soon after the transition to the DAA-era, so DAA awareness may be lower than it is currently. However, we believe the results are credible since nearly two-thirds had heard of newer HCV therapy in our study. Moreover, there are many correctional settings in the U.S. and worldwide that, to date, have had less exposure to DAA therapy [44]. Additionally, participants in this study were interviewed via convenience sampling. Because we did not have access to individuals with active behavioral or security risk our findings may not represent perceptions of all people living with HCV in this setting. Similarly, the study was conducted in a large urban U.S. jail where HCV treatment was available, so our study may not be representative of individuals who are incarcerated in other geographic areas in the U.S. or internationally, particularly where HCV treatment is not yet available. Lastly, due to the transient nature of this population we were unable to re-engage with study participants to review transcripts for comment or correction. Nevertheless, these findings further our knowledge of attitudes of people incarcerated in local jails toward HCV treatment and linkage to HCV care. The latter has particular importance for jail settings in the U.S. due to short lengths of stay, but may also have implications for prisoners on remand in international settings.

In conclusion, U.S jails are a critical node for public health intervention due to the high HCV prevalence among individuals with the greatest socioeconomic and health disparities. Our data demonstrate that the decision whether or not to be treated in jail or seek treatment upon return to the community is complex. While there is an urgency to treat this vulnerable target population, participants themselves were aware that they may have treatment interruptions and their environment pre- and post-release may not be conducive to initiating or continuing treatment. Attitudes toward HCV treatment were enmeshed in a participants’ treatment readiness, which may be hindered by social isolation, housing and employment instability, and substance use. For these reasons, patient-centered approaches to increase treatment uptake and linkage to HCV care following release from jail should focus on promoting HCV-related knowledge and awareness of the efficacy and tolerability of DAA therapy. Transitional care programs should promote social support during and after incarceration, with jail healthcare plans focused on linkage to services such as substance use disorder and mental health treatment, housing and transportation assistance, and employment services. Current policies of incarceration for individuals with substance use disorders add particular urgency to assisting with the barriers to and enhancing the facilitators of linkage to HCV care after incarceration. Without addressing HCV in the criminal justice system, we will not succeed in eliminating HCV.
